# The Role of Machine Learning in Predicting Hospital Readmissions Among General Internal Medicine Patients: A Systematic Review

**DOI:** 10.7759/cureus.84761

**Published:** 2025-05-24

**Authors:** Mukul Sharda, Saujas Sharma, Shaunak Raikar, Nathaniel Verhagen, Janavi Wagle, Ritisha Mathur, Saathvik Gowda, Sharath Kommu, Rupesh Prasad, Sanjay Bhandari, Pinky Jha

**Affiliations:** 1 Internal Medicine, Medical College of Wisconsin, Milwaukee, USA; 2 Neuroscience, University of Michigan, Ann Arbor, USA; 3 Chemical Engineering, University of Wisconsin-Madison, Madison, USA; 4 Science, Brookfield East High School, Brookfield, USA; 5 Science, Brookfield Academy, Brookfield, USA; 6 Science, Brookfield Central High School, Brookfield, USA; 7 Internal Medicine, Marshfield Clinic Health System, Marshfield, USA; 8 Internal Medicine, Aurora Sinai Medical Center, Milwaukee, USA

**Keywords:** artificial intelligence, general internal medicine, machine learning, readmission, us based

## Abstract

Hospital readmissions contribute significantly to healthcare costs. While traditional regression models for predicting 30-day readmission risk offer modest accuracy, machine learning (ML) presents an opportunity to capture complex relationships in healthcare data, potentially enhancing predictions. This review assesses the role of ML in predicting 30-day readmissions for general internal medicine admissions in the U.S. Following the Preferred Reporting Items for Systematic reviews and Meta-Analyses (PRISMA) guidelines, a literature search of PubMed (2014-2023) was conducted using the keywords "artificial intelligence," "machine learning," and "readmission." The review focused on ML models predicting readmissions in general internal medicine patients in the U.S. Nine studies were reviewed, covering conditions like acute myocardial infarction (AMI), heart failure (HF), pneumonia (PNA), chronic obstructive pulmonary disease (COPD), and other general internal medicine cases. ML models such as artificial neural networks (ANN), random forests (RF), gradient boosting, logistic regression, and natural language processing (NLP) were used. ANN and RF models outperformed traditional regression methods, while NLP-based approaches showed limited success. Subgroup modeling provided marginal improvements in predictive accuracy. In conclusion, ML offers significant potential for improving 30-day readmission predictions by overcoming the limitations of traditional models. ANN and RF are particularly effective in predicting readmissions in general internal medicine. To advance predictive capabilities, future research should refine NLP, subgroup modeling, and focus on model generalizability, integration of diverse data sources, and the development of explainable AI for clinical adoption. Addressing these challenges could transform healthcare delivery, improve patient outcomes, and reduce costs.

## Introduction and background

In 2012, the Centers for Medicare and Medicaid Services (CMS) launched the Hospital Readmissions Reduction Program (HRRP) to reduce the risk of readmission for patients hospitalized due to acute myocardial infarction (AMI), pneumonia (PNA), and heart failure (HF). In 2014, the program expanded to include elective total knee and total hip replacements as well as chronic obstructive pulmonary disease (COPD) exacerbations [1]. Despite these efforts, approximately 15% of patients discharged after acute hospitalization are readmitted within 30 days, contributing to worsened clinical outcomes and billions of dollars in healthcare costs [1]. In response, multiple initiatives have been introduced to minimize readmissions, including penalties for hospitals with excessive readmission rates among Medicare beneficiaries under the HRRP and similar programs targeting commercial insurance plans [2-5]. These efforts aim to incentivize hospitals to improve patient outcomes by reducing avoidable readmissions.

Various predictive models were developed to assess the risk of readmissions. However, the existing prediction models for hospital readmissions have generally shown limited success. Traditional regression-based models often report area under the curve (AUC) values in the range of 0.63 to 0.65, indicating a modest ability to distinguish high-risk patients from others [6,7]. In recent years, machine learning (ML) algorithms have evolved to predict hospital readmissions. They have the potential to reveal nuanced, nonlinear relationships between variables in healthcare data, a task that traditional methods, such as logistic regression, have struggled to capture. Various ML models, such as random forests (RF) and artificial neural networks (ANN), have been employed to overcome the limitations associated with traditional models and have shown modest improvements in prediction accuracy [8-10].

This review aims to critically assess the accuracy and predictability of modern ML models in predicting 30-day hospital readmissions related to general internal medicine. While systematic reviews have evaluated various ML models in predicting readmissions, few have specifically focused on general internal medicine admissions within the United States. We hypothesized that ML models, such as ANN and RF, demonstrate higher accuracy and predictive performance in identifying the 30-day hospital readmission risks for general internal medicine patients compared to traditional regression-based models by leveraging their ability to capture complex, nonlinear relationships in healthcare data.

## Review

Methods

This systematic review was conducted in accordance with the Preferred Reporting Items for Systematic Reviews and Meta-Analyses (PRISMA) 2020 guidelines [11]. Although the review was not registered in a formal protocol registry, such as the International Prospective Register of Systematic Reviews (PROSPERO), it adhered to standard methodological practices.

With the assistance of an academic librarian specializing in Health Sciences, the authors developed the search strategies for this review. We searched the PubMed database using the terms "artificial intelligence," "machine learning," and "readmission," focusing on articles published between January 1, 2014, and December 31, 2023.

Three authors (SK, SB, and PJ) searched and reviewed the studies and abstracts for inclusion in the review. Any discrepancies in the selection process were resolved through mutual discussion. Only studies meeting the following inclusion criteria were included: 1) use of at least one ML model for predicting hospital readmissions, 2) studies focused on general internal medicine patients, 3) original research, and 4) US-based.

Once relevant studies were identified, data extraction and PRISMA risk of bias assessment were performed by three authors (MS, SS, and SR) and subsequently validated by the authors SK, SB, PJ, and RP. Extracted data included the study's name and publication year, location, sample size, research objectives, the AI/ML models employed, key findings, and conclusions.

A quantitative meta-analysis was not conducted due to significant methodological heterogeneity across the included studies. Specifically, variations existed in machine learning algorithms used, outcome definitions, clinical populations, and performance metrics reported. These discrepancies in study design and outcomes prevented meaningful statistical aggregation, thus warranting a qualitative systematic review approach.

Results

**Figure 1:**
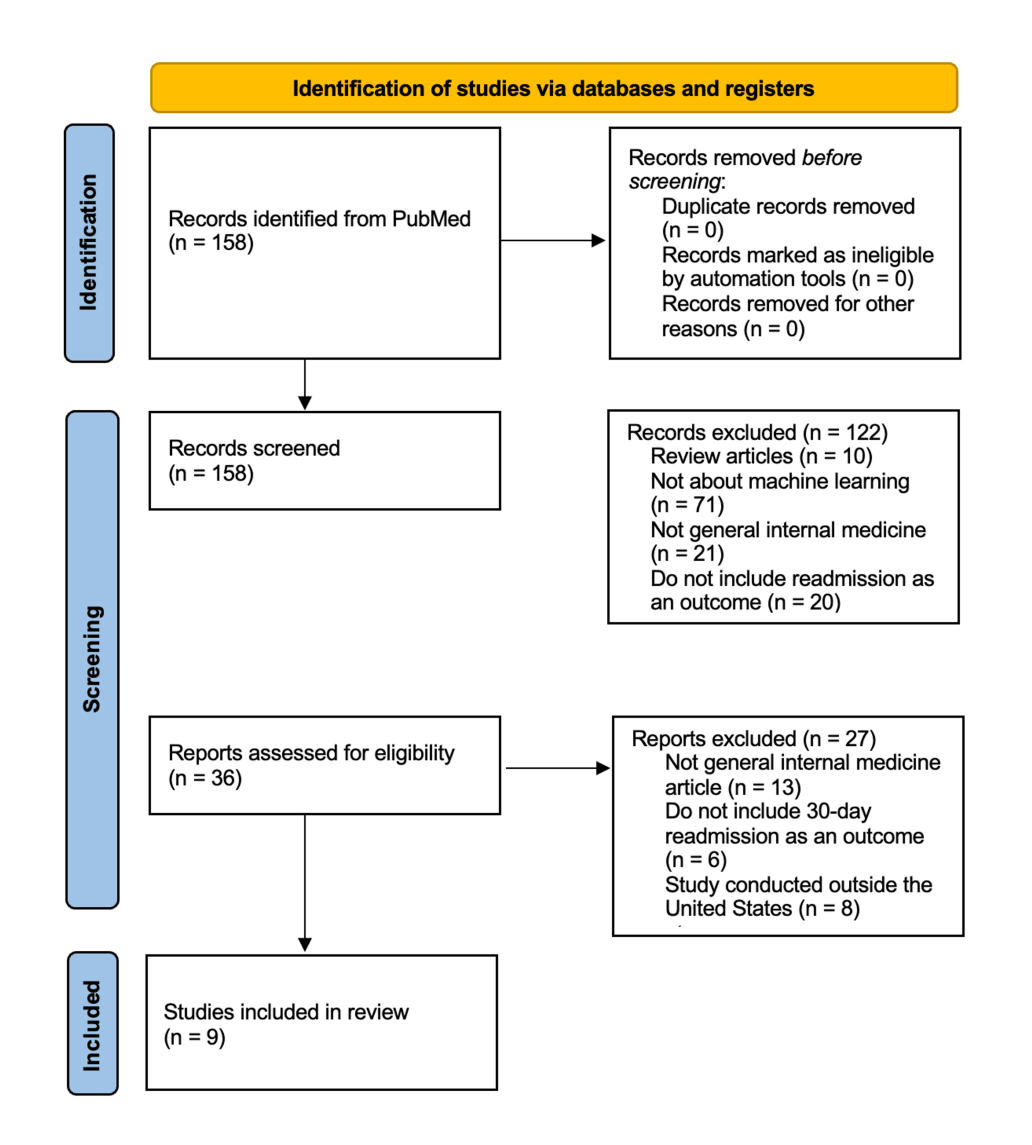
PRISMA flowchart illustrating the selection process for studies included in the review PRISMA: Preferred Reporting Items for Systematic reviews and Meta-Analyses

The initial literature search identified 158 studies. Article titles and abstracts were screened based on study inclusion criteria. One hundred twenty-two articles were excluded since they did not meet the study inclusion criteria, resulting in 36 advancing to full-text review. Data were aggregated from the final nine uniquely selected articles [9,12-19] which met the study inclusion criteria (Figure 1).

**Figure 2:**
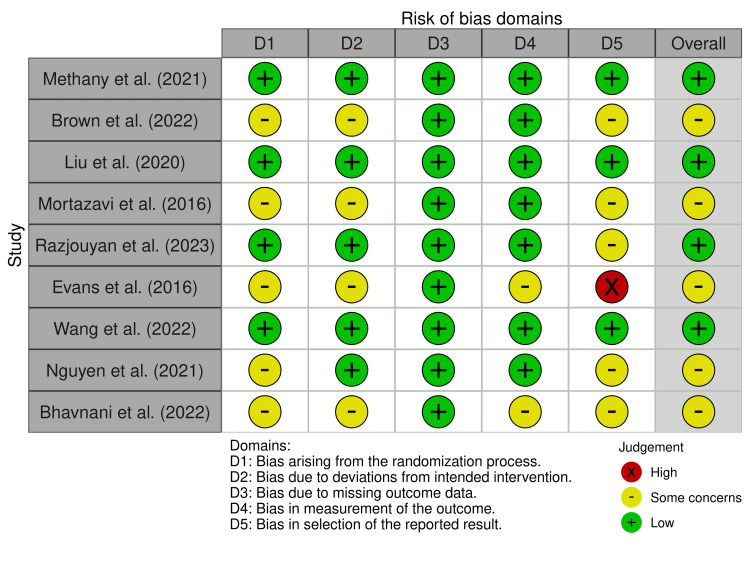
PRISMA risk of bias assessment for the included studies PRISMA: Preferred Reporting Items for Systematic reviews and Meta-Analyses [9,12-19]

Risks of bias across various domains were assessed, including those arising from the randomization process due to deviations from intended interventions, missing outcome data, the measurement of the outcome, and the selection of the reported result (Figure 2).

While the studies by Methany et al. [12], Liu et al. [14], Razjouyan et al. [15], and Wang et al. [17] were found to have a low risk of bias, the other studies had some concerns of bias (Figure 2). 

Data sources for these studies included electronic health records (EHRs) from individual institutions in five of the nine studies, large databases in three studies, and clinical trial data in one study. The predictive models analyzed across these studies were diverse, encompassing elastic net regression (two studies), least absolute shrinkage and selection operator (LASSO) regression (three studies), ridge regression (two studies), RF (four studies), gradient boosting (four studies), logistic regression (three studies), ANN (two studies), C language processing (NLP) (two studies), Poisson regression (one study), EHR-based prediction models (one study), and modeling and interpreting patient subgroups (MIPS) in one study. Table 1 provides detailed information on all the nine studies included in this review.

**Table 1:** Summary of the characteristics of the included studies, such as the study title, study location, number of participants, the aim of the study, and the AI/ML model used AI: Artificial Intelligence; AMI: Acute Myocardial Infarction; ANN: Artificial Neural Networks; AUC: Area Under Curve; AUROC: Area Under the Receiver Operating Characteristic Curve; CI: Confidence Interval; COPD: Chronic Obstructive Pulmonary Disease; EHR: Electronic Health Record; EN: Elastic Net; GB: Gradient Boosting; HF: Heart Failure; LASSO: Least Absolute Shrinkage and Selection Operator; LR: Logistic Regression; MIPS: Modeling and Interpreting Patient Subgroups; ML: Machine Learning; NSRF: Natural Language Processing–derived Social Risk Factors; NLP: Natural Language Processing; PNA: Pneumonia; pFI: Parsimonious Frailty Index; PR: Poisson Regression; RF: Random Forest; RR: Ridge Regression; SCD: Structured Clinical Data; SVM: Support Vector Machines; THA/TKA: Total Hip Arthroplasty/Total Knee Arthroplasty; VHA: Veterans Health Administration

Sr No	Study title (citation)	No. of participants	Study location	Study aim	AI/ML model used	Results	Conclusion
1	Development of electronic health record-based prediction models or 30-day readmission risk among patients hospitalized for acute myocardial infarction (Matheny et al. [12])	Total of 10187 patients; Derivation cohort: 6163 patients; Validation cohort: 4024 patients	Derivation cohort: Vanderbilt University Medical Center; Validation cohort: Dartmouth-Hitchcock Health Center	To compare multiple ML models for predicting the 30-day readmission risk in patients hospitalized for AMI using EHR derived data standardized to a common data model (model or tool development)	Parametric models: EN, LASSO, and ridge regression. Nonparametric models: RF and GB machines.	The AUROC for the derivation cohort: 0.686 to 0.695 for the parametric models and 0.686 to 0.704 for the nonparametric models. Validation cohort: 0.558 to 0.655 for parametric models and 0.606 to 0.608 for the nonparametric models.	This study developed five ML models and externally validated them to predict 30-day readmission after AMI hospitalization. These models can be deployed within an EHR using routinely collected data.
2	Information extraction from electronic health records to predict readmission following acute myocardial infarction: does natural language processing using clinical notes improve prediction of readmission? (Brown et al. [13])	A total of 10189 patients; Derivation cohort: 6165 patients; Validation cohort: 4024 patients	Derivation cohort: Vanderbilt University Medical Center; Validation cohort: Dartmouth-Hitchcock Health Center	To investigate whether the integration of social risk factors using NLP and ML can improve the prediction of 30-day readmission following an AMI.	Five ML models, three parametric: EN, LASSO, RR, and two nonparametric: RF, GB. These models were applied to three different data sets: SCD only. SCD+NSRF and NSRF main effects.	For the derivation cohort, the testing set AUROC for SCD only: 0.681 to 0.705. SCD+NSRF: 0.654 to 0.703. NSRF: 0.519 and 0.629. Similar trends observed in the validation cohort.	Negative study. Adding NSRF did not improve the prediction of hospital readmission following AMI.
3	Predicting 30-day hospital readmissions using artificial neural networks with medical code embedding (Liu et al. [14])	202,038 admissions for AMI; 303,233 admissions for HF; and 327,833 admissions for PNA	University of Michigan Medical School and affiliated departments. Data taken from the 2014 Nationwide Readmissions Database	To evaluate the effectiveness of ANN with medical code embedding in predicting 30-day hospital readmissions for AMI, HF, and PNA.	Four prediction models: hierarchical LR, GB, and two ANN models (one of which uses a novel embedding approach)	Compared to the hierarchical LR model, the ANN model improved AUC from 0.68 (95% CI 0.678–0.683) to 0.72 (95% CI 0.718–0.722) for the AMI cohort, from 0.60 (95% CI 0.592–0.597) to 0.64 (95% CI 0.635–0.639) for the HF cohort, and from 0.63 (95% CI 0.624–0.632) to 0.68 (95% CI 0.678–0.683) for the PNA cohort.	Positive study. The ANN models incorporating medical code embeddings outperformed hierarchical LR and GB models in predicting 30-day readmissions.
4	Analysis of machine learning techniques for heart failure readmissions (Mortazavi et al. [9])	Derivation cohort: 1,004 patients Validation cohort: 977 patients	Data drawn from Tele-HF trial, which enrolled 1,653 patients from 33 cardiology practices across the U.S. between 2006 and 2009	By including comprehensive clinical, socioeconomic, psychosocial, and health status data from the Tele-HF trial, this study aimed to use this wide range of data to outperform traditional models and provide more accurate predictions of readmission and mortality risks.	Traditional statistical models: LR and PR. ML models: RF, boosting, and SVM.	RF improved 30-day all-cause readmission prediction by 17.8% compared to LR. For 30-day HF readmission, RF showed a 23.2% improvement.	ML models improved discrimination and predictive range for HF readmissions compared to traditional linear models.
5	Developing a parsimonious frailty index for older, multimorbid adults with heart failure using machine learning (Razjouyan et al. [15])	37,431 patients admissions with a primary diagnosis of HF	VHA medical centers	To create a simple, efficient tool that could help clinicians assess frailty and manage HF patients at risk for adverse outcomes by identifying key predictive features from the VHA EHR’s. (Model or tool development)	LASSO, Survival RF, Unsupervised ML (K-means clustering)	Study successfully developed a pFI using only 10 variables, which demonstrated strong predictive ability for key outcomes like mortality, 30-day and 1-year emergency department visits, and hospital readmissions in older, multimorbid HF patients. pFI AUC was 0.64, which is better than other indices such as the VA Frailty Index (0.60), the Charlson Comorbidity Index (0.62), and the Elixhauser Comorbidity Index (0.62), demonstrating incremental improvement.	The pFI is a practical and efficient tool for clinical use, requiring only 10 variables.
6	Automated identification and predictive tools for high-risk heart failure patients: a pilot evaluation (Evans et al. [16])	175 patients	A 354-bed IH hospital treating high-risk HF patients in Utah/Idaho	To develop and evaluate an automated system utilizing NLP and a predictive risk model to identify hospitalized HF patients and assess their 30-day readmission and mortality risk.	NLP and Predictive Modeling, Clinical Decision Support	NLP improved the sensitivity of identifying HF patients from 82.6% to 95.3%, and the specificity from 82.7% to 97.5%.	There was no change in 30-day readmission rates. However30-day mortality was improved and increased home discharges.
7	AI-driven clinical care pathway to reduce 30-day readmission for COPD patients (Wang et al. [17])	Training sample: 332 patients; Validation sample: 172 patients	Houston Methodist Hospital	To create a simple, AI-powered tool capable of early identification of COPD patients at risk of readmission.	ANN model vs LR	The ANN model AUC: 0.683 (±0.009) on the training data, outperforming the LR model AUC: 0.640 (±0.038). On the validation dataset, the ANN model AUC: 0.77. Implementing the algorithm in Re-Admit app, along with a clinical care plan for high-risk patients, there was a 48% decline in the COPD readmission rate in the high-risk subgroup.	The Re-Admit app, powered by the ANN-based predictive model, accurately classified COPD patients at risk for 30-day readmission as early as day one of their hospital admission.
8	Man vs. machine: comparing physician vs. electronic health record-based model predictions for 30-day hospital readmissions (Nguyen et al. [18])	1,183 hospitalizations across 1,119 unique patients	Parkland Hospital in Dallas, Texas	To evaluate whether clinician predictions or an EHR-based prediction model provided more accurate forecasts for 30-day readmissions, and to explore whether a combined approach could yield better outcomes.	EHR-based model	This combined approach achieved a higher C-statistic of 0.70 (95% CI 0.67–0.74, p=0.001 compared to clinicians alone, and p=0.002 compared to the EHR model alone).	Combining human and machine insights provided a superior method for predicting 30-day hospital readmissions compared to using either approach independently.
9	A framework for modeling and interpreting patient subgroups applied to hospital readmission: a visual analytical approach (Bhavnani et al. [19])	COPD: 29,016 patients; CHF: 51,550 patients; and THA/TKA: 16,498 patients	Data taken from Medicare insurance claims data set	To develop and assess a novel framework for MIPS to predict hospital readmission risks (Model or tool development)	MIPS framework – Visual analytical model, classification model, prediction model	COPD prediction model: The standard model had a C-statistic 0.624 (95% CI 0.617-0.631), nearly identical to the hierarchical model’s C-statistic of 0.625 (95% CI 0.618-0.632); CHF prediction model: The standard model had a C-statistic of 0.600 (95% CI 0.595-0.605), and the hierarchical model also had a C-statistic of 0.600 (95% CI 0.595-0.606); THA/TKA prediction model: Both the standard and hierarchical models had a C-statistic of 0.638 (95% CI 0.629-0.646 for the standard model; 95% CI 0.629-0.647 for the hierarchical model)	The predictive accuracy gains from incorporating subgroup membership were minimal.

The study by Methany et al. (2021) evaluated five ML models for predicting 30-day readmission risk in patients hospitalized for AMI using standardized EHR data [12]. The models, including elastic net, LASSO, ridge regression, RF, and gradient boosting machines, were developed with a derivation cohort of 6,165 patients from Vanderbilt University Medical Center and validated with 4,024 patients from Dartmouth-Hitchcock Health Center [12]. The research highlighted the importance of robust model calibration and discrimination while addressing challenges such as inconsistent data across institutions and ensuring model portability. The findings demonstrated the potential of EHR-based predictive models to support clinical decision-making and improve post-AMI care by integrating these tools into real-world clinical settings. While this study developed ML models, it did not address the benefit of ML models over traditional rule-based or linear models in predicting readmissions.

The study by Brown et al. evaluated whether incorporating NLP-derived social risk factors into ML models could improve the prediction of 30-day readmission risk following AMI [13]. Using data from Vanderbilt University Medical Center and Dartmouth-Hitchcock Health Center, the study applied the rule-based NLP tool Moonstone to analyze clinical notes for seven social risk factors. ML models, including elastic net, LASSO, ridge regression, RF, and gradient boosting machines, were trained on structured clinical data (SCD) alone, SCD combined with NLP-derived factors and NLP-derived factors alone [13]. The addition of social risk factors did not significantly improve model performance, with area under the receiver operating characteristic (AUROC) scores similar across all datasets [13]. The study concluded that current NLP methods and data sources may be insufficient for enhancing readmission prediction, emphasizing the need for novel approaches to improve accuracy.

The study by Liu et al. assessed the use of ANN with medical code embedding to predict 30-day hospital readmissions for AMI, HF and PNA using the 2014 Nationwide Readmissions Database [14]. Four models were evaluated: hierarchical logistic regression, gradient boosting (XGBoost), and two ANN models, with one using ICD-9 codes as latent variables learned through GloVe embeddings [14]. The ANN model with medical code embeddings significantly outperformed other models, achieving the highest AUC scores across all cohorts: 0.72 for AMI, 0.64 for HF, and 0.68 for PNA, compared to lower scores for logistic regression and gradient boosting [14]. The study demonstrated that embedding medical codes into a latent space enhances predictive accuracy, marking a positive step toward improved readmission risk prediction across diverse conditions.

The study by Mortazavi et al. evaluated ML techniques to predict 30-day and 180-day readmissions or mortality in HF patients using data from the Telemonitoring to Improve Heart Failure Outcomes (Tele-HF) trial, which included 1,653 patients and 472 variables. ML models such as RF, boosting, and support vector machines (SVM) were compared to traditional logistic and Poisson regression models [9]. The RF model demonstrated notable improvements, enhancing 30-day all-cause readmission prediction accuracy by 17.8% and HF readmissions by 23.2% compared to logistic regression [9]. By capturing complex, nonlinear interactions among variables, ML models provided superior predictive accuracy, enabling better identification of high-risk patients for targeted interventions. The study highlighted the potential of advanced analytics in improving care and emphasized the importance of refining methods and data for even greater prediction capabilities.

The study by Razjouyan et al. developed and validated a parsimonious frailty index (pFI) for older, multimorbid adults with HF using ML to predict outcomes such as mortality, emergency department visits, and readmissions [15]. Using data from 37,431 patients in Veterans Health Administration (VHA) medical centers, ML methods, including LASSO, survival random forest (Surv-RF), and K-means clustering, reduced an initial set of 76 features to a concise set of 10 highly predictive variables [15]. The pFI demonstrated strong predictive accuracy (AUC 0.64) and outperformed traditional indices in identifying high-risk patients, with the severely frail group showing the highest mortality and healthcare utilization rates [15]. The study concluded that the pFI is a simple, efficient tool for real-time frailty assessment and targeted intervention planning, improving outcomes for frail HF patients.

The study by Evans et al. evaluated an automated system using NLP and predictive risk models to identify and assess the 30-day readmission and mortality risk for high-risk HF patients [16]. Conducted at an Intermountain Healthcare hospital, the study involved 175 patients and used the Intermountain Risk Score (IMRS) to calculate risk based on clinical data. The NLP model improved the patient identification sensitivity (82.6% to 95.3%) and specificity (82.7% to 97.5%) for patients with HF, with a positive predictive value of 97.45% [16]. While 30-day readmission rates were unchanged, significant improvements were observed in mortality reduction and increased discharges to home care. Integrating these tools into clinical workflows facilitated earlier identification and better risk stratification, improving patient outcomes and care coordination through enhanced discharge planning and timely interventions.

The study by Wang et al. developed and validated an ANN-based tool to identify patients with COPD at high risk for 30-day readmission, using data from Houston Methodist Hospital [17]. Based on four parameters-recent inpatient admissions, medications on day one, insurance status, and the Rothman Index-the ANN model outperformed traditional logistic regression, achieving an AUC of 0.77 on validation data [17]. Implemented through the "Re-Admit" app, the model enabled early risk stratification and targeted interventions, such as in-hospital teaching and care-transition planning, resulting in a 48% reduction in readmission rates for high-risk patients [17]. The study underscored the potential of AI-driven tools like the Re-Admit app to enhance clinical decision-making, improve patient outcomes, and lower healthcare costs.

The study by Nguyen et al. compared the ability of clinicians and an EHR-based model to predict 30-day hospital readmissions, analyzing 1,183 hospitalizations at Parkland Hospital in Dallas. Both approaches demonstrated similar predictive accuracy (C-statistic 0.66), but their predictions showed modest concordance, with clinicians skewing toward lower risk and the EHR model toward higher risk [18]. A combined "human-plus-machine" model, integrating clinician assessments and the EHR score, achieved superior performance (C-statistic 0.70) and better stratified patients by risk, ranging from 12% in low-risk to 34% in high-risk groups [18]. The findings highlighted that combining clinician insights with ML enhances predictive accuracy, suggesting that future strategies should integrate both approaches to optimize patient outcomes.

The study by Bhavani et al. introduced the Modeling and Interpreting Patient Subgroups (MIPS) framework to predict hospital readmission risks for patients with COPD, CHF, and total hip arthroplasty/total knee arthroplasty (THA/TKA) by identifying subgroups based on co-occurring comorbidities [19]. The framework utilized visual analytics, subgroup classification, and hierarchical prediction modeling. While subgroup classifications were accurate and statistically significant, incorporating subgroup information into prediction models resulted in minimal improvements in predictive accuracy compared to standard models (C-statistics for both approaches were similar across all conditions) [19]. The findings suggested that comorbidities alone are insufficient predictors of readmissions and underscore the need for more advanced subgroup modeling techniques to enhance interpretability, improve predictions, and ultimately reduce readmission risks.

Discussion

ML is emerging as a transformative tool in healthcare, enabling predictive modeling and risk stratification that can improve clinical decision-making and patient outcomes. Various ML models were developed for the prediction of various healthcare parameters, including disease diagnosis, prognosis, and outcomes [20-26]. Similarly, there are several models developed for predicting hospital readmissions, and there are various review articles investigating the same. However, review articles focusing exclusively on ML models and hospital readmissions are very few [27-30]. To our knowledge, ours is the first article that exclusively focuses on hospital readmissions among general internal medicine patients with data exclusively involving the US population.

The nine studies reviewed in our study illustrate the strengths and limitations of various ML approaches applied to predicting hospital readmissions across diverse conditions. Positive results from ANN and RF models emphasize the potential of ML to enhance prediction accuracy, while the limited benefit observed with NLP and visual-analytic models highlights the importance of optimizing ML techniques for specific applications.

Studies using ANN models for AMI, HF, and PNA demonstrated the efficacy of embedding medical codes into latent spaces, enabling better representation of clinical features and achieving the highest predictive accuracy (AUCs of 0.72, 0.64, and 0.68, respectively) [14]. This approach leverages the strength of deep learning in capturing nonlinear relationships and complex patterns in large datasets. Similarly, the ANN-based "Re-Admit" tool for patients with COPD showed significant improvements in identifying high-risk patients, achieving an AUC of 0.77 and reducing 30-day readmissions by 48% through targeted interventions [17]. These findings highlight ANN’s adaptability and precision in diverse clinical contexts when trained on well-structured datasets with meaningful variables. Along similar lines, a review on ML and readmissions by Huang et al. showed that neural networks (NN), in addition to boosted tree-based models, performed strongly in predicting readmissions [27]. Similarly, Mahmoudi et al. showed that NN, in addition to the RF model, was the most popular ML method for predicting readmission [28].

RF models also demonstrated their strength in predicting HF outcomes by capturing complex, nonlinear interactions among variables. In one study, RF outperformed traditional regression models, improving prediction accuracy for 30-day all-cause readmissions by 17.8% and HF-specific readmissions by 23.2% [9]. In contrast, studies evaluating NLP-based models for AMI and HF failed to show significant benefits in improving prediction accuracy [13,16]. While NLP successfully extracted social risk factors from clinical notes, these factors did not enhance model performance when integrated with structured clinical data [13]. These results suggest that current NLP methods may lack the granularity or contextual understanding necessary to extract predictive value from unstructured text. Future advancements in NLP algorithms and access to richer data sources may be needed to realize their full potential in clinical risk prediction. Similarly, visual-analytic models used in the MIPS framework for COPD, CHF, and post-THA/TKA readmissions offered limited predictive improvement over standard models [19]. Although these models excelled in identifying statistically significant patient subgroups based on comorbidities, the subgroups did not translate into meaningful gains in predictive accuracy.

A key takeaway from these studies is that the success of ML models depends on their ability to integrate and analyze high-quality, relevant data. Positive models such as ANN and RF excelled by leveraging structured datasets with clear variables, while NLP and visual-analytic approaches struggled due to limitations in data sources and model design. Additionally, hybrid approaches, such as combining clinician insights with EHR-based predictions, have shown promise, as seen in the "human-plus-machine" model, which outperformed standalone methods for predicting readmissions.

These studies also highlight the importance of tailoring ML approaches to specific clinical problems. For example, ANN models were particularly effective for diseases like AMI and COPD with well-defined risk factors, while RF models proved suitable for HF, where complex interactions among numerous variables play a significant role. On the other hand, NLP models faced challenges in extracting actionable insights from unstructured data, underscoring the need for context-aware algorithms and more robust text-processing techniques.

As ML continues to advance, its potential to transform the prediction of hospital readmissions is immense. Future efforts should focus on developing models with both high predictive accuracy and generalizability across diverse patient populations and healthcare settings. Achieving this will require addressing challenges such as variability in data quality, completeness, and representation across institutions. Portable models, which can adapt to different healthcare environments without extensive retraining, will be critical for widespread adoption. Moreover, integrating additional data sources, such as real-time physiological monitoring, social determinants of health, and patient-reported outcomes, could significantly enhance model performance. Advances in explainable artificial intelligence (XAI) will also be vital, ensuring that clinicians can interpret and trust ML predictions to guide clinical decisions effectively. Collaboration between healthcare providers, data scientists, and policymakers will be necessary to standardize data practices, validate models rigorously, and align predictions with meaningful clinical actions. By addressing these priorities, ML has the potential to not only predict hospital readmissions with greater precision but also to identify actionable interventions, reduce healthcare costs, and improve patient outcomes on a global scale.

Limitations

Our review has various limitations. First, only nine studies were assessed in this review. This is because of the strict inclusion criteria, which included only readmissions related to general internal medicine and studies within the USA. While this allowed for a more focused and detailed analysis, it limits the generalizability of our findings across other specialties or international contexts. Second, because various studies analyzed different diagnoses and different ML models were used in these studies, we were unable to find ML models common to a larger group of studies to give more discrete conclusions. Third, the portability between different EHRs of the prediction models evaluated here is limited, and it is unclear if the models which showed positive results can be utilized across various EHR systems. Fourth, our review did not delve deep into the various individual factors of an ML model that contributed to its better performance compared to others.

Finally, an important limitation of our review is the exclusive reliance on the PubMed database for literature search. While PubMed is an extensive and authoritative biomedical resource, restricting our search to a single database may have inadvertently excluded relevant studies indexed in other databases, such as Embase, Scopus, or Web of Science. Consequently, this limitation may have introduced publication bias and could have impacted the comprehensiveness and generalizability of our findings. Future studies should incorporate broader database searches to enhance coverage and ensure a more exhaustive evaluation of the available literature.

## Conclusions

In conclusion, the application of ML in healthcare offers significant promise for improving clinical decision-making and patient outcomes in predicting hospital readmissions across various conditions. Positive results from ANN and RF models in predicting hospital readmissions related to general internal medicine highlight the potential of these advanced techniques to handle complex, high-dimensional data and deliver more accurate predictions. However, the challenges faced by NLP and visual-analytic models underscore the importance of refining algorithms and improving data quality to fully realize their potential. As healthcare continues to evolve, integrating ML models with clinical workflows, optimizing their accuracy, and ensuring their practical applicability will be crucial in driving improvements in patient care and health system efficiency. Continued advancements in AI, data integration, and collaborative efforts between clinicians and data scientists will be key to achieving the next generation of healthcare innovation.
